# The relationship between family function and career adaptability among telecommunications employees: the chain mediation effect of social support and general self-efficacy

**DOI:** 10.3389/fpsyg.2026.1811209

**Published:** 2026-07-16

**Authors:** Jinghua Zhu, Xinqing Xu, Zhenyu Pan, Jingjing Song, Hanzhong Zhang, Jiangang Shao, Yalei Li, Liping Jia

**Affiliations:** 1 Department of Psychology, Shandong Second Medical University, Weifang, Shandong, China; 2 Network Information Center, Shandong Second Medical University, Weifang, Shandong, China; 3 Department of Public Health, Shandong Second Medical University, Weifang, Shandong, China; 4 Faculty of Psychology, Tianjin Normal University, Tianjin, China

**Keywords:** career adaptability, family function, general self-efficacy, social support, telecommunications employees

## Abstract

**Objective:**

Career adaptability is an important personal resource for employees' career development. Among telecommunications employees, the relationship between family function as an environmental resource and career adaptability requires further study. Based on Career construction theory and Conservation of resources theory, this study used mediation analysis to explore the influence mechanism of family function on career adaptability and the roles of social support and general self-efficacy.

**Methods:**

An online survey was conducted using the Family Assessment Device, the Career Adapt-Abilities Scale, the Social Support Rating Scale, and the General Self-Efficacy Scale among 10,516 employees of a telecommunications company in Shandong, China, yielding 7,625 valid responses.

**Results:**

Social support and general self-efficacy are mediating factors between family function and career adaptability, accounting for 11.325% and 33.493% of the total effect, respectively. Furthermore, social support and general self-efficacy had a chain mediating effect between family function and career adaptability, accounting for 12.530% of the total effect.

**Conclusion:**

This study identified a resource conversion pathway linking family function to career adaptability among telecommunications employees, in which social support and general self-efficacy jointly played a chain mediating role. The findings provide empirical evidence for the combined application of Career construction theory and Conservation of resources theory in this occupational group.

## 1 Introduction

The telecommunications industry is undergoing rapid development, with frequent technological updates and iterations. In this context, telecommunications employees' work is highly dependent on technical competence, requiring them to adapt to rapid technological changes. At the same time, residents' demand for telecommunications and network services is increasing (Statistics, 2025). In daily work, telecommunications employees need to respond to customer needs in real time, handle service complaints, and meet performance and time requirements. Therefore, telecommunications companies represent a typical high-pressure work environment. Recent studies have also found that many employees in this industry face work stress and its negative consequences (Liu et al., 2024a; Xu et al., 2024). Existing research on various high-pressure occupations has consistently shown that work stress is associated with lower job satisfaction and higher turnover intention (Chen and Qi, 2022; Norful et al., 2024), which further intensifies the talent crisis in organizations.

Career adaptability is an important psychosocial resource that individuals possess when facing complex career environments (Savickas, 2013), and it helps employees effectively cope with career challenges in high-pressure environments. Previous studies have shown that career adaptability can effectively help individuals overcome difficulties and maintain higher performance and stability during organizational change (Chen et al., 2020). Among employees in manufacturing enterprises, higher career adaptability helps them actively cope with uncertainty in organizational change and career development, and show stronger career control, career curiosity, and confidence in facing challenges (Gao et al., 2019). It can also help employees deal with environmental conflicts and stress while maintaining work-life balance (Zhou and Zheng, 2022). In contrast, insufficient career adaptability is associated with higher turnover intention (Yu and Zheng, 2013). However, existing research has focused more on the role of career adaptability, while paying insufficient attention to its influencing factors.

Career construction theory (CCT) suggests that individuals promote their career development through adaptive behaviors and resources (Rudolph et al., 2019). The development of career adaptability depends on whether the social environment can provide continuous support to buffer external challenges (Savickas and Porfeli, 2012; Rudolph et al., 2017). Among various environmental factors, family is the most important microsystem for individuals. Family is closely connected with individuals in terms of time and space and has strong emotional bonds, so it is considered an important source influencing career adaptability (Chen et al., 2020). However, how family function promotes employees' career adaptability remains unclear.

Conservation of resources theory (COR) provides a key perspective. This theory suggests that individuals tend to acquire, retain, and protect resources, and that they build and protect existing resources through resource investment (Hobfoll, 2001). Within this framework, family function is regarded as a relatively stable environmental resource. It refers to the overall ability of the family to provide support, coordination, and emotional responsiveness to its members at the system level, and represents the basic operational characteristics and deeper attributes through which the family promotes members' development (Miller et al., 1985; Cao et al., 2013).

Previous studies have shown that good family function is associated with higher levels of individual adaptation (Boyes et al., 2023). It also helps employees manage work–family boundaries more effectively and promotes the effective accumulation and conversion of resources (Jia and Su, 2020; Gu et al., 2024). Individuals with poorer family function are more likely to experience impaired mental health and reduced work quality (Tao et al., 2021). At the same time, work–family supportive behaviors have been shown to reduce work–family conflict (Lei et al., 2023). They can increase job satisfaction and subjective well-being (Gragnano et al., 2020), and may also promote job performance (Br Ginting et al., 2024). Therefore, in the telecommunications industry, we propose that family function may influence employees' career adaptability.

COR theory further suggests that family function may promote the development of career adaptability by increasing energy resources and personal resources. Among these, energy resources refer to resources that help individuals obtain and maintain other resources, such as social support. Social support refers to all forms of support that provide material and psychological resources to help individuals cope with stress. From the perspective of the social support buffering model, support from significant others can play a protective role under stress and help individuals cope with challenges more effectively (Cohen and Wills, 1985). The better the family function, the more likely family members are to provide stable emotional responsiveness and problem-solving support. This can increase the level of social support available to the individual. Many studies have examined social support as a mediator in the association between family function and related outcomes. Their results consistently show a significant positive relationship between family function and social support (Li et al., 2024). In addition, family support may enhance perceived support through processes such as promoting work–family balance and alleviating stress experiences (Ren et al., 2022; Le et al., 2023). On this basis, it is necessary to further examine whether social support, as an energy resource, can promote the development of career adaptability. Existing studies have shown that social support is significantly associated with higher career adaptability, and this finding is consistent across different studies and samples (Rencber and Paşaoglu Baş, 2023; Stead et al., 2025). Moreover, workplace social support provides a resource base for employees' support and confidence when facing change and challenges, and is an important factor in enhancing employees' adaptability (Karatepe and Olugbade, 2017; Lee et al., 2021). Therefore, in the telecommunications industry, we propose that social support mediates the relationship between family function and career adaptability.

In COR theory, personal resources are also important factors that promote career adaptability. They refer to individuals' own abilities and traits, such as general self-efficacy. Different from domain-specific self-efficacy, general self-efficacy reflects individuals' confidence in their overall coping ability when facing challenging situations (Schwarzer, 1997; Liu et al., 2024b). Studies have shown that family function, as a relatively stable source of environmental resources, promotes the formation and maintenance of general self-efficacy (Zakiei et al., 2020). Specifically, individuals with higher levels of family support and stronger work–family compensation experiences tend to report higher general self-efficacy (Erum et al., 2020; Hao et al., 2025). Meanwhile, general self-efficacy is closely related to career adaptability and positively predicts career adaptability among university students (Zhang, 2019). It is also associated with higher levels of career adaptability in working populations (Vashisht et al., 2023). Based on these findings, in the telecommunications industry, we propose that general self-efficacy may mediate the relationship between family function and career adaptability.

In the meantime, studies have found that social support is significantly and positively associated with general self-efficacy across different groups (Li, 2021; Samulowitz et al., 2022). The more social support individuals receive, the stronger their confidence when facing stress and work (Zhang et al., 2023). In contrast, insufficient social support is associated with lower general self-efficacy. Individuals with low self-efficacy are more likely to exhibit avoidant, passive, or helpless coping responses to work and life challenges, and are also more likely to be embedded in social environments with insufficient support (Wensu et al., 2021). This suggests that a resource conversion process may exist between energy resources and personal resources. As an important source of environmental resources, family function may increase individuals' social support. After individuals obtain social support, it may promote the formation and maintenance of general self-efficacy. General self-efficacy is closely related to higher levels of career adaptability. Based on this, we propose that, among telecommunications employees, family function may influence career adaptability through social support and general self-efficacy through a chain mediation pathway.

From a theoretical perspective, CCT emphasizes the influence of family on career adaptability as an adaptive outcome. COR regards family function as an environmental resource, social support as an energy resource, general self-efficacy as a personal resource, and career adaptability as a psychosocial resource, and emphasizes the transformation process among these resources. By integrating these two theories, family function may influence career adaptability through the resource transformation pathway of social support and general self-efficacy. Based on this, we propose that, among telecommunications employees, family function may affect career adaptability through the chain mediating roles of social support and general self-efficacy.

In summary, career adaptability is an important psychosocial resource for individuals to cope with stress and career uncertainty. For employees, it is essential for maintaining stable development and achieving continuous career growth. However, the resource transformation pathway through which family function influences career adaptability still needs to be further clarified. Therefore, based on the integrated perspective of CCT and COR theory, this study constructed a chain mediation model to examine whether family function affects career adaptability among telecommunications employees through social support and general self-efficacy, providing theoretical support and practical guidance for improving the career adaptability of employees in the telecommunications industry. This study proposes the following hypotheses: (1) family function has an influence on employees' career adaptability; (2) social support and general self-efficacy separately mediate the relationship between family function and career adaptability; and (3) social support and general self-efficacy play a chain mediating role between family function and career adaptability.

## 2 Materials and methods

### 2.1 Participants

This study used stratified random sampling to conduct an online survey among employees from several city-level branches of a telecommunications company in Shandong Province, China. All participants took part voluntarily, and all questionnaires were completed anonymously. The survey was administered from January to February 2024. A total of 10,516 questionnaires were collected. After excluding outliers and incomplete questionnaires based on Mahalanobis distance and response consistency, 7,625 valid questionnaires were retained. The valid response rate was 72.51%. Males accounted for 51.80% of the sample, and outsourced employees accounted for 74.11%. This study was approved by the Ethics Committee of Shandong Second Medical University (Approval No. 2023YX118).

### 2.2 Materials

#### 2.2.1 Family function

We used the Family Assessment Device (FAD) to assess employees' family function comprehensively. The scale was developed by Miller et al. (1985) based on the McMaster Model of Family Functioning. The Chinese version was revised by Li et al. (2013). It includes 60 items. The FAD includes six specific dimensions. In addition, it contains a general functioning dimension, which represents the overall level of family function. The scale uses a 4-point Likert format, ranging from 1 (not like my family at all) to 4 (very much like my family). After score transformation, higher scores indicate better family function. The FAD has good reliability and validity in China. In this study, Cronbach's α was 0.933.

#### 2.2.2 Career adaptability

The Career Adapt-Abilities Scale (CAAS) was developed by Savickas and Porfeli (2012). The Chinese version was translated and revised by Hou et al. (2012). It includes four dimensions and contains 24 items. The scale uses a 5-point Likert format, ranging from 1 (not strong) to 5 (very strong). Higher scores indicate higher levels of career adaptability. In this study, Cronbach's α was 0.975.

#### 2.2.3 Social support

We used the Social Support Rating Scale (SSRS) developed by Xiao (1994) to assess social support. The scale includes three dimensions and contains 10 items. Higher scores indicate higher levels of social support. The SSRS has been widely used in China. In this study, Cronbach's α was 0.745.

#### 2.2.4 General self-efficacy

The General Self-Efficacy Scale (GSES) was developed by Chen et al. (2001). The Chinese version was translated and revised by Wang et al. (2001). It includes 10 items. The scale uses a 4-point Likert format, ranging from 1 (not at all true) to 4 (exactly true). Higher scores indicate higher levels of general self-efficacy. In this study, Cronbach's α was 0.923.

The indicators included in each variable are summarized in Table 1.

**Table 1:** Dimensions included in each variable.

Variable	Scale	Dimensions	Example item	Number of items
Family function	Family Assessment Device (FAD)	Problem solving	After our family tries to solve a problem, we usually discuss whether the problem has been resolved.	6
		Communication	When someone in the family is upset, the others know why he or she is upset.	9
		Roles	We are sure that family members have fulfilled their respective family responsibilities.	11
		Affective responsiveness	We express tenderness and affection.	6
		Affective involvement	If someone gets into trouble, the others become overly concerned.	7
		Behavior control	We have rules for dealing with dangerous situations.	9
		General functioning	We are able to make decisions about how to solve problems.	12
Career adaptability	Career Adapt-Abilities Scale (CAAS)	Career concern	Thinking about what my future will be like.	6
		Career control	Making decisions by myself.	6
		Career curiosity	Looking for opportunities to grow.	6
		Career confidence	Being responsible for doing things well.	6
Social support	Social Support Rating Scale (SSRS)	Objective support	What were the sources of financial support and practical help you received when you encountered emergencies or difficulties in the past?	3
		Subjective support	How many close friends do you have who can provide support and help?	4
		Support utilization	How do you usually talk to others when you are troubled?	3
General self-efficacy	General Self-Efficacy Scale (GSES)	General self-efficacy	If I try my best, I can always solve problems.	10

### 2.3 Data analysis

IBM SPSS 26.0 was used for data processing and analysis. The analyses included common method bias, descriptive statistics, difference tests, and correlation analysis. For the mediation analysis, all data were standardized. Path modeling was conducted using the PROCESS 4.1 plug-in developed by Hayes (2012). Model 6 was selected for chain mediation analysis. The bootstrap method was used with 5,000 resamples to test the significance of the indirect effects (α = 0.05).

## 3 Results

### 3.1 Common method bias

Because self-report measures were used to collect the data, common method bias may be present. Therefore, Harman's single-factor test was conducted. The results showed that 16 factors had eigenvalues greater than 1. The first factor explained 24.45% of the variance, which was below the 40% threshold. Therefore, this study did not show serious common method bias, and subsequent analyses could be conducted. We further used the unmeasured latent method construct (ULMC) approach to examine common method bias. The results showed that, compared with the single-factor model, the two-factor model showed changes in model fit indices of ΔCFI = 0.053, ΔTLI = 0.054, ΔRMSEA = −0.022, and ΔSRMR = −0.018. According to the recommendation proposed by Wen et al. (2018), if the increases in CFI and TLI after adding the method factor are less than 0.10, and the decreases in RMSEA and SRMR are less than 0.05, this indicates that there is no serious common method bias. Therefore, this study did not show serious common method bias.

### 3.2 Descriptive statistics and correlation analysis

To better explore the mediation pathway, we used independent-samples *t* tests and one-way ANOVA. We examined the effects of demographic factors, including gender, age, and years of service, with career adaptability as the dependent variable. The results showed that male employees had significantly higher career adaptability scores (M = 84.880) than female employees (M = 77.416; *t* = 15.064, *p* < 0.001). In addition, career adaptability differed significantly by age (*F* = 14.545, *p* < 0.001), years of service (*F* = 19.785, *p* < 0.001), and labor relationship (*t* = −8.351, *p* < 0.001). These variables will be used as control variables in the subsequent analysis. See Table 2.

**Table 2:** Demographic information test.

Variables	Categories	Career adaptability	Family function	General self-efficacy	Social support
Age	≤ 28	84.455	176.200	26.256	40.509
	29~35	80.715	171.929	25.615	42.597
	36~45	79.958	171.394	25.876	43.810
	≥46	82.473	174.544	26.921	45.379
	F/t	14.545^***^	22.385^***^	10.338^***^	58.186^***^
Gender	Male	84.880	172.702	26.753	43.011
	Female	77.416	172.862	25.187	43.152
	F/t	15.064^***^	−0.355	10.720^***^	−0.637
Years of service	≤ 5	84.137	174.812	26.42	42.401
	5~10	79.720	170.254	25.512	42.236
	10~15	78.568	170.611	25.263	42.689
	15~20	80.447	172.351	25.992	44.386
	≥20	82.343	175.881	26.99	45.259
	F/t	19.785^***^	24.033^***^	16.497^***^	24.081^***^
Labor relationship	Outsourced employees	80.086	171.471	25.735	42.938
	Regular employees	84.758	176.612	26.762	43.492
	F/t	−8.351^***^	−9.693^***^	−6.231^***^	−2.179>^*^

^*^p < 0.05.

^***^p < 0.001.

The correlation results are shown in Table 3. There are significant correlations among all variables.

**Table 3:** Pearson correlation coefficients among the variables.

Variable	M ±SD	1	2	3	4
1	172.779 ± 19.788	1			
2	43.079 ± 9.676	0.457^**^	1		
3	25.997 ± 6.420	0.378^**^	0.345^**^	1	
4	81.275 ± 21.937	0.419^**^	0.347^**^	0.622^**^	1

^**^p < 0.01; 1 Family Function; 2. Social Support; 3. General Self-Efficacy; 4. Career Adaptability.

### 3.3 Mediating effect analysis

**Figure 1:**
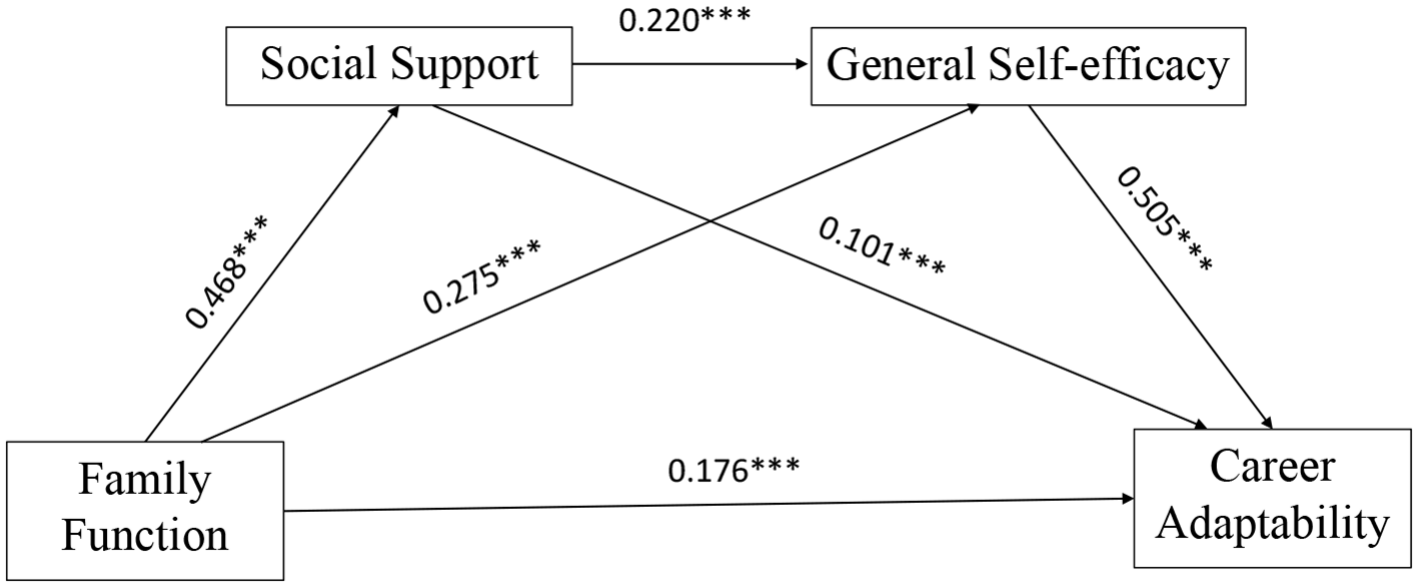
Mediating effect diagram of social support and general self-efficacy. ***p < 0.001.

After all data were standardized, PROCESS Model 6 was used. Family function was set as the independent variable, and career adaptability was set as the dependent variable. Social support and general self-efficacy were specified as mediators. Age, gender, years of service, and labor relationship were included as control variables in the chain mediation model. The results showed that family function had a direct positive effect on career adaptability, with a regression coefficient of 0.415 (*t* = 40.418, *p* < 0.001). After adding the mediators, family function showed a significant direct positive effect on career adaptability (β = 0.176, *t* = 17.505, *p* < 0.001). Family function positively predicted social support (β = 0.468, *t* = 46.561, *p* < 0.001) and general self-efficacy (β = 0.275, *t* = 23.462, *p* < 0.001). Social support significantly and positively predicted general self-efficacy (β = 0.220, *t* = 18.682, *p* < 0.001), and also positively predicted career adaptability (β = 0.101, *t* = 10.176, *p* < 0.001). General self-efficacy positively predicted career adaptability (β = 0.505, *t* = 53.294, *p* < 0.001). See Table 4 and Figure 1.

**Table 4:** Regression analysis between variables.

Regression equation		Goodness of fit index		Coefficient significance	
Dependent variable	Independent variable	*R^2^*	F	β	*t*
Social support	Family function	0.489	479.678	0.468	46.561^***^
	Age			0.226	13.598^***^
	Gender			0.031	1.549
	Years of service			−0.036	−3.222^**^
	Labor relationship			−0.078	−3.304^**^
General self-efficacy	Family function	0.443	310.305	0.275	23.462^***^
	Social support			0.220	18.682^***^
	Age			0.005	0.307
	Gender			−0.243	−11.682^***^
	Years of service			−0.012	−1.046
	Labor relationship			0.057	2.361^*^
Career adaptability	Family function	0.669	884.538	0.176	17.505^***^
	Social support			0.101	10.176^***^
	General self-efficacy			0.505	53.294^***^
	Age			−0.053	−3.763^***^
	Gender			−0.218	−12.520^***^
	Years of service			−0.017	−1.860
	Labor relationship			0.078	3.884^***^

^*^p < 0.05.

^**^p < 0.01.

^***^p < 0.001.

The results indicated that the mediating effect of social support was significant (β = 0.047, 95% CI [0.038, 0.057]). The mediating effect of general self-efficacy was significant [β = 0.139, 95% CI (0.125, 0.153)]. The overall chain mediating effect was significant [β = 0.052, 95% CI (0.045, 0.059)]. See Table 5.

**Table 5:** Direct, indirect, and total effects.

Path	Effect (95%CI)	SE	Relative effect (%)
Family function → social support → career adaptability	0.047 (0.038, 0.057)	0.005	11.325%
Family function → general self-efficacy → career adaptability	0.139 (0.125, 0.152)	0.006	33.493%
Family function → social support → general self-efficacy → career adaptability	0.052 (0.045, 0.059)	0.003	12.530%
Indirect effect	0.239 (0.223, 0.255)	0.008	57.590%
Direct effect	0.176 (0.156, 0.196)	0.010	42.409%
Total effect	0.415 (0.395, 0.435)	0.010	–

## 4 Discussion

This study adopted a perspective of environmental to individual psychosocial resource conversion. Based on Career construction theory (CCT) and Conservation of resources theory (COR), it examined how family function among telecommunications employees influences the development of career adaptability through social support and general self-efficacy.

The results showed that family function plays an important role in the development of career adaptability among telecommunications employees. Family function significantly and positively predicted employees' career adaptability. This finding supports our hypothesis. That is, higher family function was associated with stronger ability to cope with external demands. This is largely consistent with previous research (Hao, 2022). This finding is also consistent with Career Construction Theory (CCT). CCT emphasizes that career development is achieved through continuous interaction with external resource environments. A cohesive family environment can help individuals obtain support and reduce stress. It can convert environmental resources into resources for coping with external challenges (Miller et al., 2000; Fan et al., 2024). Employees can obtain understanding and support from a good family environment and convert these resources into positive psychosocial resources, which may help them maintain work–family balance (Thompson et al., 2021).

In light of the resource conversion pathway from family function to career adaptability, firstly, the results showed that social support had a significant mediating effect between family function and career adaptability. This finding supports our hypothesis. Employees with better family function typically receive more social support, which as an energy resource further promotes the development of career adaptability. This is consistent with previous studies. Individuals with better family function can obtain emotional support, understanding, and help from family members (Jiao et al., 2024). Such support can help employees enhance their career adaptability (Wang et al., 2023). The results also showed a significant positive association between social support and career adaptability. In adolescent samples, social support has been found to act as a protective resource that shapes career confidence and promotes adaptation to future career development (Li et al., 2023). Similarly, we confirmed this association among telecommunications employees.

At the same time, the results showed that general self-efficacy, as a personal resource, mediated the relationship between family function and career adaptability. The research results support the hypothesis. This is consistent with previous findings in university student samples (Zakiei et al., 2020). Positive daily behaviors and lifestyles within the family are an effective way to cultivate general self-efficacy (Locke, 1997). They enhance individuals' confidence and ability to cope with work challenges. Even in working populations, a good family environment still has a significant influence on the formation and maintenance of self-efficacy. The results showed that general self-efficacy positively predicted career adaptability. This suggests that individuals with higher general self-efficacy typically have stronger adaptability, more confidence in solving problems, and a greater tendency to actively seek help from others (Rahman et al., 2020; Stead et al., 2022). Although this study used general self-efficacy in a broad sense rather than occupational self-efficacy, both are important factors influencing career adaptability.

In addition, this study further found that social support and general self-efficacy played a chain mediating role between family function and career adaptability. This finding supports our hypothesis. From the perspective of COR theory, career adaptability, as a psychosocial resource, is influenced by the transformation between environmental resources and personal resources. When family function, as an environmental resource, is stronger, it may provide better problem-solving and communication abilities for individuals, thereby improving employees' social support. This helps individuals cope with stress and challenges in daily life and work, further enhances their general self-efficacy, and strengthens their confidence and ability to deal with career challenges, ultimately improving their career adaptability. These results further indicate that the integrated perspective of CCT and COR helps clarify the relationship between family function and career adaptability, as well as the resource transformation pathway between them.

This study has several practical implications. The findings show that family function is associated with career adaptability among telecommunications employees, and that social support and general self-efficacy play mediating roles in this relationship. Although organizations may find it difficult to directly change employees' family interaction patterns, they can provide employee assistance programs, family communication guidance, and stress management services to help employees better deal with work pressure and family communication problems. These measures may enhance employees' experiences of being understood, responded to, and supported in both family and work contexts, thereby improving their perceived social support. At the same time, organizations can also improve employees' general self-efficacy through supervisor support, peer support, career development feedback, and staged work goal management. Supervisors can provide clearer task feedback and career advice, while peer support can help employees gain experience and emotional support when facing difficulties. Through continuous external support and successful experiences, employees may develop stronger coping confidence. These measures may improve employees' perceived social support and general self-efficacy, and further promote their career adaptability.

This study also has some limitations. First, as a cross-sectional study, it is difficult to fully examine causal relationships and dynamic processes among the variables. Future research can use longitudinal follow up designs to further test the dynamic relationships among family function, social support, general self-efficacy, and career adaptability.

Second, all participants were recruited from one telecommunications company in Shandong Province, China. This may limit the generalizability of the findings. Therefore, caution is needed when applying the results to telecommunications employees in other regions, other telecommunications companies with different management practices, or employees in other high-pressure industries. Future studies could include multi-region, multi-company, and cross-industry samples to test whether the present findings are stable across different occupational and organizational contexts.

This study did not further examine the role of gender. However, previous studies have found that male and female employees face different forms of occupational stress in the workplace (Pace and Sciotto, 2022; Hu, 2025), and that the resources available to them may also differ. Future research could consider exploring how gender differences influence career adaptability among employees.

In addition, this study measured employees' psychological constructs only through self-report, which may be influenced by social desirability. Future studies can combine self-report measures with structured interviews to examine selected employees' family and work situations in depth as well as their career adaptability.

## 5 Conclusions

In summary, social support mediated the relationship between family function and career adaptability among telecommunications employees (11.3%); general self-efficacy also played a mediating role (33.5%); and social support and general self-efficacy had a chain mediating effect between family function and career adaptability (12.5%). These indirect paths explained more variance. Building on CCT, which emphasizes the role of the family, this study further specified family as family function. Based on COR theory, this study also identified the resource transformation pathway between family function and career adaptability, indicating that this relationship mainly operates through social support and general self-efficacy. This study provides evidence for the combined application of Career construction theory and Conservation of resources theory to telecommunications employees. These findings suggest that improving the career adaptability of telecommunications employees requires joint efforts from both families and organizations. Families can provide stable emotional support and a positive communication environment. At the same time, organizations can create a supportive work atmosphere and strengthen resource provision, thereby enhancing employees' perceived social support and general self-efficacy. These measures can jointly promote the career adaptability of telecommunications employees.

## Data Availability

The raw data supporting the conclusions of this article will be made available by the authors, without undue reservation.
